# Optimal scanning concentration of MR imaging for tumor-bearing nude mice with SPIO-shRNA molecular probe

**DOI:** 10.1038/s41598-020-73923-2

**Published:** 2020-10-29

**Authors:** Liqiang Zhang, Xinyi Yang, Ming Wen

**Affiliations:** Department of Radiology, The First Affiliated Hospital of Chongqing Medical University, Chongqing, 400016 China

**Keywords:** RNAi, Ovarian cancer

## Abstract

The objective of this study is to investigate the signal changes and optimal scanning concentration of MRI in tumor tissues of tumor-bearing nude mice by SPIO-shRNA molecular probes. 30 BALB/c tumor-bearing nude mice were randomly divided into 5 groups with 6 mice in each group. At the given scanning time (before and 27 h after injection), the caudal vein was respectively injected with iron content of 6 mg·kg^−1^, 12 mg·kg^−1^, 18 mg·kg^−1^, 24 mg·kg^−1^, and 30 mg·kg^−1^, and MR examination was simultaneously performed to measure signal intensity changes of tumor tissue and contralateral muscle tissue in each concentration group. After each examination above, the nude mice were sacrificed immediately, and the tumor and muscle tissues were removed for HE and Prussian blue staining,and observed under light microscope. Nude mice in 6 mg, 12 mg and 18 mg groups all survived after probe injection, but some nude mice died in 24 mg and 30 mg groups after probe injection or during scanning. The signal changes of T2WI and T2*WI sequences were the most obvious in MR scanning sequences. Compared with other groups, the signal intensity of the tumor tissue in 18 mg, 24 mg and 30 mg groups were most obvious (P < 0.05), while the 18 mg, 24 mg and 30 mg groups reached no statistical difference (P > 0.05 ); HE staining indicated that structural disorder of tumor tissue as well as increase of nuclear atypia. Prussian Blue staining showed that blue-stained iron particles were present in each experimental group,and the most densely distributed were in 18 mg,24 mg and 30 mg groups. Tumor tissue could be well labeled with SPIO-shRNA molecular probes, and the optimal MR scanning concentration (iron content) is 18 mg·kg^−1^.

## Introduction

Emerging molecular imaging is a technique for imaging the pathology and physiological changes of the body from the molecular or cellular level. It has great development and application prospects in the early diagnosis, precise positioning and monitoring of therapeutic effects of diseases. Gene therapy based on RNAi (RNA interference) technology has attracted more and more attention due to its unique high efficiency, specificity and non-invasiveness, and has become a new direction for the treatment of malignant tumors. In the study of molecular imaging, the preparation of molecular probes is the main link^1^. Based on the theory of RNA interference (RNAi)^2–4^, the present study combined molecular imaging and gene therapy with SPIO as the MRI negative contrast agent, PLL as the gene carrier, and ovarian cancer EGFR as the gene target, in order to design the shRNA (short hairpin RNA), and construct the specific SPIO-sh RNA molecules probe (SPIO-shRNA, hereinafter referred to as probe). This was a dual-functional molecular probe that integrated the diagnosis and treatment. In the early stage, the pharmacokinetics of this probe and the distribution of main organs in vivo have been verified^5,6^. On this basis, the probe was prepared with different concentrations, and injected into living tumor-bearing nude mice for MRI. These were compared with the pathological results to obtain the best concentration of MR.

## Materials and methods

The present study was approved by the Clinical research ethics committee of The First Affiliated Hospital of Chongqing Medical University, including any relevant details. All experiments were performed in accordance with the relevant guidelines and regulations.

### Main materials and equipment

The probe was prepared by the research group, who won national invention patents (Patent No.: ZL201410064217.6). SKOV3 tumor cell strain (Shanghai Institute of Cell Library, Chinese Academy of Sciences); RPMI-1640 cell culture medium and fetal bovine serum (GIBCO, USA); 0.25% trypsin (HYCLONE, USA); PBS phosphate buffer; CO_2_ thermostatic cell incubator (Thermo Forma, USA); 30 BALB/c nude mice, female, 4–6 weeks old, weighing 22 ± 3 g, purchase and feeding by the animal experimental center of Medical University Of Chongqing (Provincial Key Laboratory); Pentobarbital sodium (Merck, Germany); Nuclear Fast Red (Wuhan Boster); K4Fe (Shanghai Biological Engineering Co. Ltd.), 3.0T superconducting MR imager and wrist coil (GE, USA).

### Experimental methods and steps

#### SKOV3 tumor cell line culture and establishment of tumor-bearing nude mice model

Referring to the previous mature methods of the research group^7–10^, SKOV3 ovarian cancer cell lines were routinely cultured in medium containing 10% fetal bovine serum and RPMI-1640 (constant temperature at 37 °C, 5% CO_2_ in an incubator). The selected logarithmic phase growth cells were digested with 0.25% trypsin, collected into a 10-ml centrifuge tube, centrifuged at 800 r/min for five minutes, and diluted with serum-free antibiotic-free cell suspension to a cell concentration of 5 × 10^7^/ml for reserve. Then, 30 BALB/c nude mice were subcutaneously inoculated with SKOV3 tumor cell suspension of approximately 0.2 ml (containing approximately 1 × 10^7^ cells) in the region near the left thigh of each posterior back, and were routinely fed. The mental state, diet and tumor growth of nude mice was observed and recorded at a fixed time every day (10 o'clock in the morning). At 20 ± 5 days after inoculation, a tumor diameter of greater than 1.0 cm was detected, suggesting that the tumor-bearing nude mice were successfully modeled, and could be used for further experiments.

#### MR scanning of tumor-bearing nude mice in vivo after probe injection

##### Time selection of MRI after probe injection

The selection of scanning time was different in the literature^11,12^. After multiple discussions and repeated experiments, the present research group determined that the scanning time of the present experiment was 27 h after the probe injection.

##### Determination of probe injection concentration and experimental grouping

With reference to the relevant literature^13–16^, and combined with the previous pharmacokinetics and toxicological data^5,6^, the probe concentration (in terms of iron content) used for the experiments were 6 mg·kg^−1^, 12 mg·kg^−1^, 18 mg·kg^−1^, 24 mg·kg^−1^ and 30 mg·kg^−1^. Correspondingly, these tumor-bearing nude mice were randomly divided into five groups, with six mice each group.

##### Parameter setting of MRI and selection of scanning sequence

Considering that the scanning time was 27 h after probe injection, if the tumor-bearing nude mice are continuously anesthetized during the period from pre-injection to the end of the MRI, the experimental effect may be affected, because this does not conform to the living conditions of the animals. Hence, MRI is selected as the control before probe injection. After the end of the scan, each group was injected with different concentrations of probes, returned to the animal experiment center for conventional sub-box feeding, and started again the MRI scan at 27 h after the tail vein probe injection. The positions, parameters and sequences of the two scans were consistent. Before each MRI scan, 2% pentobarbital sodium anesthesia (dose of 0.1 ml/20 g) was injected into the abdominal cavity of tumor-bearing nude mice, placed on a self-made foam board, and fixed in a prone position with medical adhesive. After the respiratory rate decreased and the respiratory was smooth (in order to reduce the interference of respiratory artifacts to the MRI), this was placed in the wrist coil for MRI axial and coronal scanning. On the basis of literatures^17–20^, after the repeated pre-experiments and appropriate adjustments, the final parameters for the experiments were, as follows: (1) T1WI fast spin echo sequence (FSE): pulse sequence repetition time/echo time (TE/TR) 85/300 MinFull, coronal FOV 11 cm, axial FOV 6 cm, excitation times = 3, layer thickness = 2 mm, pitch = 0.5 mm, matrix 320 × 224, and flip angle = 20 degrees; (2) T2WI FSE:TE/TR 85/600 ms, FOV 6 cm, incentive times = 3, layer thickness = 2 mm, matrix 320 × 256, flip angle = 17 degrees; (3) T2*WI gradient echo (GRE): TE/TR 300 ms/MinFull, FOV 6 cm, motivation times = 3, layer thickness = 2 mm, pitch = 0.5 mm, matrix 320 × 256, flip angle = 20 degrees.

##### MRI acquisition and analysis

The scanning images were collected and transmitted to a GE AW4.6 workstation, in order to measure the signal intensity of the largest level of the tumor and the contralateral muscle tissue. In order to avoid errors, the region of interest (ROI) figure was defined as a square cursor, with an area of 20 mm^2^. Then, this was measured for three times, and its average value was recorded.

##### Pathological section and staining

After the second MR scan, the nude mice were immediately sacrificed by intraperitoneal injection of an excess of 2% pentobarbital sodium. The tumor tissues and tumor-bearing contralateral muscle tissues were removed and immersed in 10% buffered formalin solution for 48 h. Then, the paraffin-embedded sections were stained with H&E and Prussian blue, respectively, and observed under a light microscope.

##### Statistical methods

The results were analyzed by the means of the mean ± standard deviation. ANOVA was carried out among groups with different concentrations of SPSS 18.0. LSD analysis was used for the multiple comparisons among groups, in which P < 0.05 was considered statistically significant.

## Results

### MRI results in nude mice

None of the nude mice died before the probe injection. After the probe injection, one mouse immediately died in the 30 mg group. After the end of the MRI scan, one mouse died in both the 24 mg group and 30 mg group (heat preservation and other protective measures were taken to simulate feeding conditions as far as possible during the experiment). The other 27 nude mice survived.

#### Signal intensity changes of FSE sequence on T1WI

**Figure 1:**
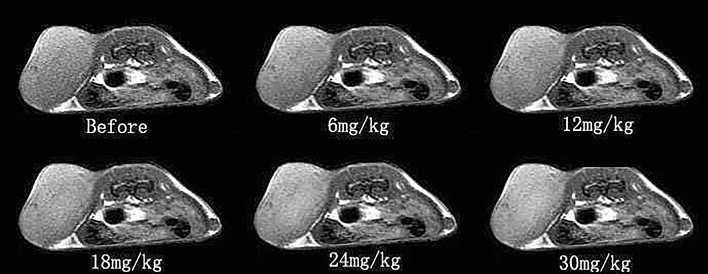
The diagram of the signal intensity T1WI FSE in tumor tissues after injection with the molecular probe at different concentrations (axial).

The signal intensity changes of tumor tissues in the different concentration groups had an abnormal signal intensity, when compared with that before probe injection, and the difference was statistically significant (P < 0.05). As shown in Table 1 and Fig. 1, the 18 mg, 24 mg and 30 mg groups were more significantly different, when compared to the 6 mg and 12 mg groups, but there was no significant difference among the 18 mg, 24 mg and 30 mg groups. The signal changes were not significant in the contralateral muscle group after probe injection, which was not statistically significant, when compared with that before injection.

**Table 1:** Signal intensity of T1WI FSE in tumor tissues and contralateral muscle tissues at different concentrations ($\overline{X}$  ± S ).

Group	Signal intensity					
	Before	6 mg	12 mg	18 mg	24 mg	30 mg
Tumor tissue	732 ± 35	777 ± 29*	798 ± 32*	843 ± 28*	865 ± 33*	876 ± 41*
Contralateral muscle tissue	588 ± 31	613 ± 24	621 ± 44	634 ± 21	625 ± 29	619 ± 26

*P < 0.05 vs. before injection (n = 30).

#### Signal intensity changes of FSE sequence on T2WI

**Figure 2:**
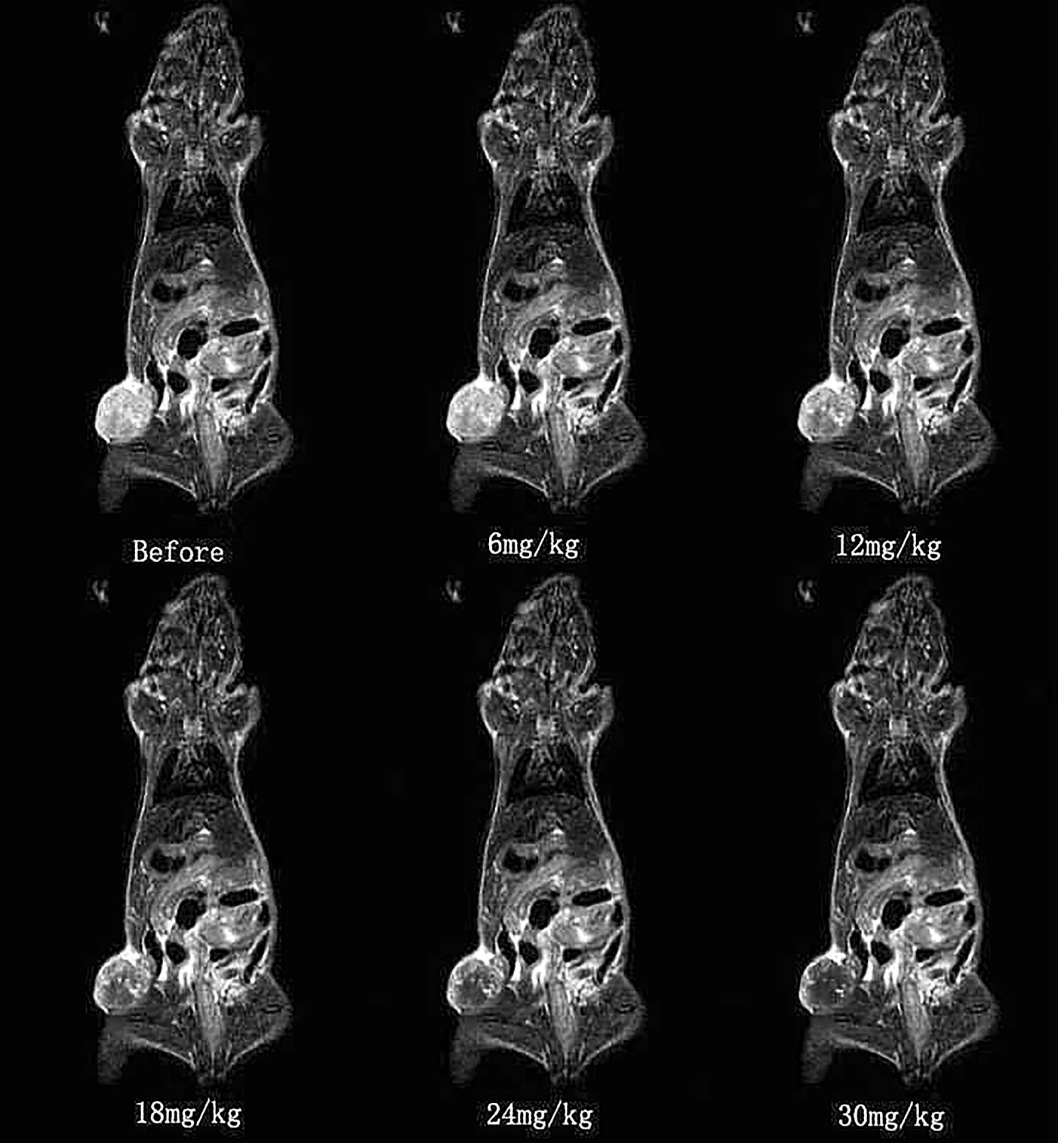
The diagram of signal intensity T2WI FSE in tumor tissues after injection with the molecular probe at different concentrations (coronal).

The signal intensity of tumor tissues in the different concentration groups changed after the injection of the probe, when compared with that before injection, and the difference was statistically significant difference (P < 0.05). After analyzing the data in Table 2 and Fig. 2, the signal intensity of tumor tissues in the 18 mg, 24 mg and 30 mg groups was more significant than that in the 6 mg and 12 mg groups, when compared with that before injection, but there was no statistical significant difference among the 18 mg, 24 mg and 30 mg groups. The signal intensity of muscle tissues in the contralateral corresponding area did not obviously change after the probe injection, but the difference was not statistically significant between each concentration group, when compared with that before the injection.

**Table 2:** Signal intensity of T2WI FSE in tumor tissues and contralateral muscle tissues at different concentrations ($\overline{X}$ ± S ).

Group	Signal intensity					
	Before	6 mg	12 mg	18 mg	24 mg	30 mg
Tumor tissue	566 ± 40	479 ± 31*	358 ± 33*	247 ± 43**	229 ± 39**	214 ± 29**
Contralateral muscle tissue	218 ± 22	203 ± 12	216 ± 18	207 ± 19	194 ± 21	199 ± 17

*P < 0.05 vs. before injection; **P < 0.001 vs. before injection (n = 30).

#### The changes of T2*WI signal intensity

**Figure 3:**
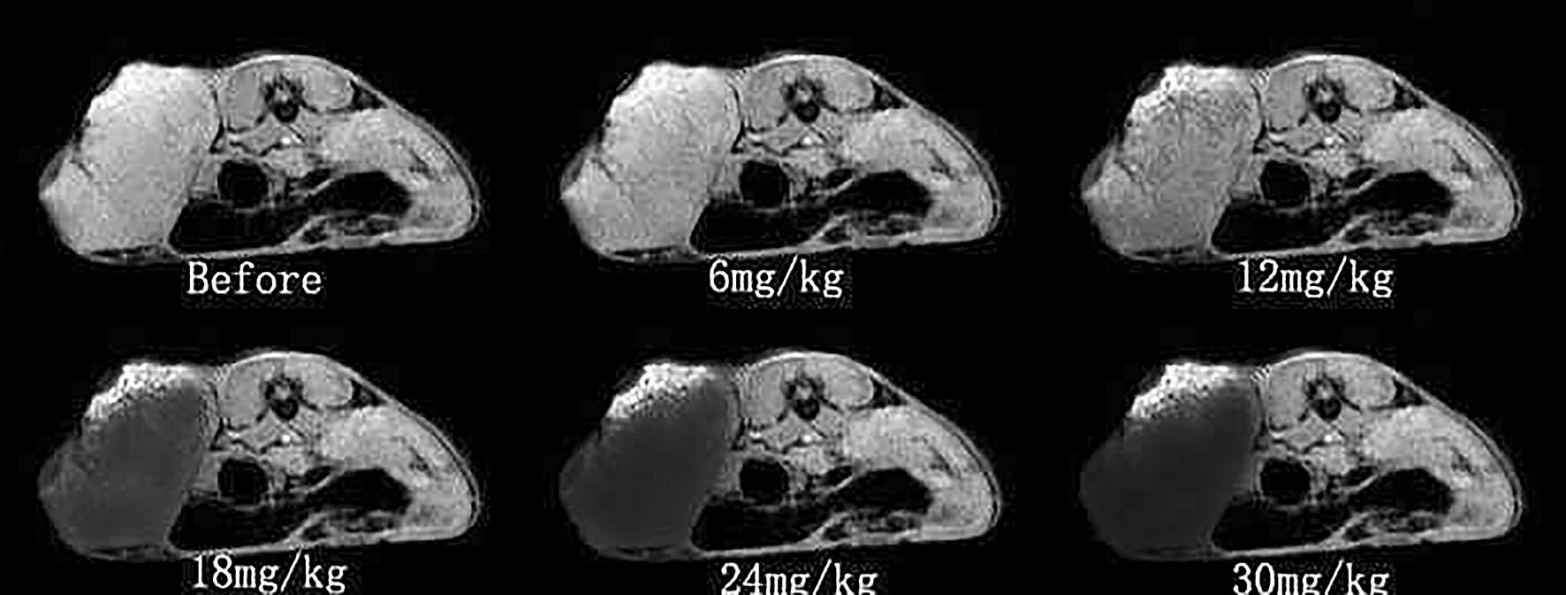
Diagram of signal intensity T2*WI in tumor tissues after injection with the molecular probe at different concentrations (axial).

After the injection of the probe, the tumor tissues in different concentration groups changed in terms of signal intensity, when compared with the pre-injection, and the difference was statistically significant (P < 0.05). After the analysis of the data in Table 3 and Fig. 3, the signal intensity of tumor tissue changes were more significant in the 18 mg, 24 mg and 30 mg groups, when compared to the 6 mg and 12 mg groups, but there was no statistical significant difference among the 18 mg, 24 mg and 30 mg groups. The contralateral area of muscle tissue signal intensity did not change after the probe injection in each concentration group, when compared to that before injection, but the difference was not statistically significant. The general change trend of the tumor was similar to that of T2WI.

**Table 3:** Signal intensity of T2*WI in tumor tissues and contralateral muscle tissues at different concentrations ($\overline{X}$ ± S).

Group	Signal intensity					
	Before	6 mg	12 mg	18 mg	24 mg	30 mg
Tumor tissue	536 ± 23	478 ± 29*	355 ± 35*	288 ± 15**	304 ± 24**	299 ± 18**
Contralateral muscle tissue	561 ± 28	510 ± 21	511 ± 22	506 ± 30	488 ± 29	492 ± 32

*P < 0.05 vs. before injection; **P < 0.001 vs. before injection (n = 30).

### Pathological results

**Figure 4:**
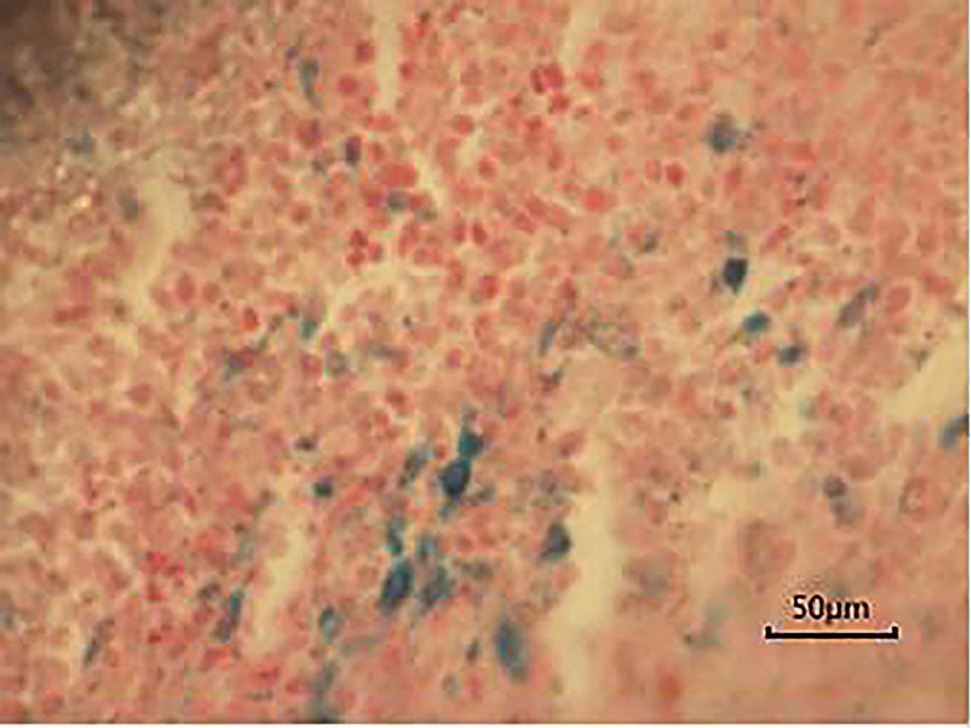
H&E straining images (magnification × 400).

**Figure 5:**
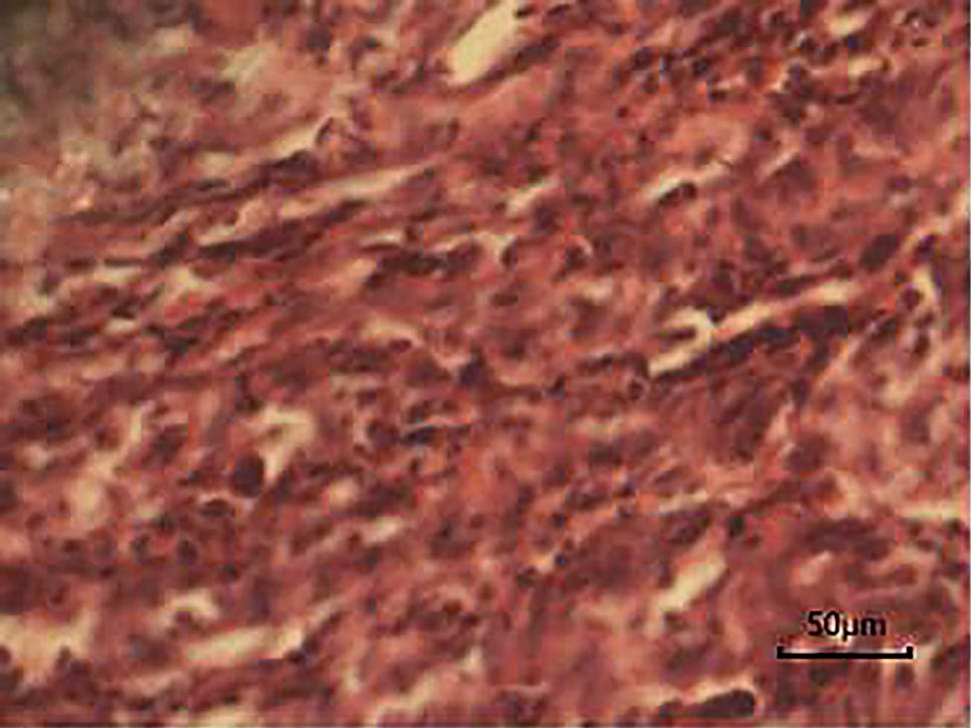
Prussian blue straining images (magnification × 400).

As shown in Fig. 4, the H&E staining results of tumor tissues in all concentration groups were similar, showing a disorder in tumor tissue structure, irregular shape and size of tumor cells, more nuclear atypia, and some mitotic phase changes, while the muscle tissues had no obvious signs, such as degeneration and necrosis. The Prussian blue staining revealed that there were no blue-stained particles in tumor tissues in each concentration group before probe injection, and that blue-stained particles could be observed in tumor tissues in all concentration groups after probe injection. When the probe concentration was 18 mg·kg^−1^, 24 mg·kg^−1^ and 30 mg·kg^−1^, there were more blue-stained particles (Fig. 5), while no blue-stained particles were found in muscle tissues in the control group.

## Discussion

Aiming at the high expression of epidermal growth factor receptor (EGFR) in ovarian cancer, the investigators linked the dextran modified SPIO with polylysine PLL to form the SPIO-PLL complex, and successfully constructed the SPIO-shRNA molecular probe for short hairpin RNA (shRNA) in epidermal growth factor receptor (EGFR)^21^. According to the RNA interference theory, endonuclease cleaves the dsRNA fragments produced during cell transcription into several small fragments of shRNA, and binds to the ribozyme complexes to silence the targeted mRNA homologous to shRNA (i.e. the epidermal growth factor receptor is overexpressed in ovarian cancer), thereby inhibiting the expression of ovarian cancer protein and causing tumor cell death^22,23^. Compared with antisense gene fragments, small fragments of shRNA have higher specificity and stability, and have a more complete inhibitory effect on tumor tissues^24,25^. In addition, the SPIO (Fe_3_O_4_/Fe_2_O_3_) contained in this probe can produce a magnetic field effect, shorten the relaxation time of the tissue, and cause low signal changes in the T2WI and T2*WI images during the MRI scan^11,26^. Therefore, theoretically, the probe prepared by combining the specificity of RNA interference with SPIO sensitivity is expected to specifically and early diagnose ovarian cancer at the molecular level. Compared with the SPIO probes that have been researched, in a previous work, the investigators combined molecular imaging and the advantages of RNA interference technology to synthesize molecular probes for the epidermal growth factor receptor highly expressed in ovarian cancer (double Function SPIO-shRNA molecular probe), which can integrate diagnosis and treatment, The results of the preliminary experiments confirmed that the probe had low cytotoxicity, and within a certain range of iron concentration, this had no significant effect on cell viability.

The results of previous experiments and related literature reports^27,28^ revealed that the probe, after injection at 24 h, mainly accumulated in the living liver and spleen, which consisted with the reticuloendothelial system and consensus phagocytosis, and decreased the signal in the T2*WI sequence of the MRI scan. With the increase in dose of the probe, the signal intensity of the liver and spleen decreased by gradient. These results provides a basis for setting the concentration gradient in this experiment. The analysis of scanning images and signal values of the above concentration groups revealed that the signal intensity decreased in tumor tissues with the probe injection, and this also confirms the former experimental results. In addition, some nude mice in the 24 mg group and 30 mg group immediately presented with the dead phenomenon after probe injection or during subsequent scanning. It was speculated that the concentration of the probe was too high, that is, nude mice could not tolerate a probe concentration of above 24 mg, suggesting that 24 mg and 30 mg were not ideal probe concentrations. The subsequent experimental results also revealed that the tumor tissue, when the probe concentration was 18 mg·kg^−1^, slightly increased on the T1WI, while showing low signal changes on the T2WI and T2*WI. The probe concentration was within the tolerance range of nude mice, and the change in signal intensity was more remarkable, when compared to the other groups, which could be distinguished by the naked eye. Hence, the optimal probe concentration for the MRI scans of tumor-bearing nude mice in vivo was 18 mg·kg^−1^. However, there was no significant change in the T1WI, T2WI and T2*WI signals in the contralateral areas, and the change trend was not the same as that in the tumor tissues. When the signal difference was obvious, the images could be well-distinguished. The pathological results also demonstrated that the expression of blue-stained iron particles was abundant in tumor tissues, and that only a few iron particles were stained in the liver and spleen, which were consistent with the observation results of the MRI. It can be inferred that the nanoscale molecular probe prepared by the research group (average particle size = 7.37 nm)^29^ can mostly escape the phagocytosis of the reticuloendothelial system, penetrate blood vessels and tissues spaces, and successfully enrich in tumor tissues, thereby achieving the intended purpose of the present experiment^30^_._

Due to the limitations of experimental equipment and various conditions, the interval of the probe injection concentration was large, and there may be more accurate probe concentrations during the period, which needs to be further improved in subsequent experiments. In the subsequent experiments, the research group would further explore the optimal scanning sequence and anti-tumor dose of the probe.

## Data Availability

The data used to support the findings of the study are available from the corresponding author upon request.
